# Health information-seeking behavior in stroke patients and its relationship with Behavioral decision-making: a latent profile analysis

**DOI:** 10.3389/fneur.2025.1683198

**Published:** 2025-12-03

**Authors:** Ze-run Zhao, Meng Yang, Juan-juan Feng, Yu-meng Lan, Juan-ping Zhong, Wei-ping Li, Xing-lei Wang, Xin-man Dou

**Affiliations:** 1 School of Nursing, Lanzhou University, Lanzhou, China; 2 Vascular Access Clinic, Lanzhou University Second Hospital, Lanzhou, China; 3 Department of Cardiovascular, Lanzhou University Second Hospital, Lanzhou, China; 4 Department of Nursing, Lanzhou University Second Hospital, Lanzhou, China

**Keywords:** stroke, health information-seeking behavior, behavioral decision-making, influencing factors, latent profile analysis

## Abstract

**Objective:**

This study explored latent profiles of Health Information-Seeking Behavior (HISB) among stroke patients and analyzed its influencing factors.

**Methods:**

In this cross-sectional study, 311 stroke participants from two tertiary care hospitals in Gansu Province, China, were recruited between January and May 2025 using convenience sampling. Data were collected using a general information questionnaire, the Health Information-Seeking Behavior Scale, and the Health Behavior Decision-Making Assessment Scale for Stroke Patients. Latent profile analysis (LPA) was employed to identify distinct HISB profiles.

**Results:**

Three latent profiles were identified: the high-demand low-barrier positive group, the moderate-balanced group, and the low-demand high-barrier negative group. Key predictors of profile membership included age, education level, monthly personal income, and the presence of comorbid chronic diseases.

**Conclusion:**

The identification of three distinct HISB trait types provides an evidence-based foundation for developing personalized health education and tailored decision support interventions. Healthcare professionals can leverage this classification system to customize communication strategies for patients with different traits, deliver tiered information support, and ultimately empower patients to achieve better health behaviors and health outcomes.

## 1 Introduction

Stroke represents a clinical syndrome arising from a group of vascular risk factors. Data from the 2021 Global Burden of Disease(GBD) Study indicate that stroke was the third most common GBD level 3 cause of death in 2021, accounting for 10.7% of all deaths. It was also the fourth leading cause of disability-adjusted life years (DALYs), representing 5.6% of total DALYs (1). In 2021, there were 93.8 million prevalent cases of stroke globally, with 11.9 million new cases occurring annually (1). China bears the most significant stroke burden globally (2). In 2021, the number of stroke patients in China accounted for 28.1% of the global total. Furthermore, it is projected that by 2050, the number of stroke patients will reach 34.27 million. As a chronic disease, stroke imposes a substantial health and economic burden on patients, their families, and society (3).

Research both domestically and internationally has reached a consensus that stroke is preventable and controllable and that about 84% of strokes are associated with correctable risk factors, such as behavioral, metabolic, psychosocial, and environmental factors (4). Behavioral change interventions are the most direct and effective interventions for controlling stroke risk factors and preventing morbidity and recurrence (5). Studies indicate that comprehensive behavioral interventions such as promoting healthy diets, regular exercise, and smoking cessation can reduce stroke incidence by 18 to 29%, often yielding greater overall benefits than drug interventions alone (6). A Brazilian study trained community health workers to enhance public awareness of stroke risks and promote behavioral changes, significantly improving participants’ lifestyles and quality of life (7). Thus, with behavioral change at its core, implementing multidimensional health management for stroke patients can effectively reduce the disability rate, mortality rate, disease burden, and recurrence risk of stroke and ultimately realize the overall improvement of the patient’s quality of life and functional independence (8).

With the development of modern economic society and mobile Internet communication technology, health information has become a crucial factor influencing people’s behavior and social activities in contemporary society. It has gradually evolved into a key variable influencing individual health behaviors (9, 10). On the one hand, by actively acquiring health information, individuals can systematically improve their knowledge of disease-related information, thereby significantly enhancing their ability to assess and recognize disease risk. On the other hand, health behaviors are influenced by how individuals cognitively process information and how information is presented. Patients make health behavior decisions by evaluating the efficacy of the queried health information and then implementing scientifically based health behaviors to prevent or control diseases (11, 12). Improving health information access behaviors in stroke patients may positively affect changing health behaviors and promoting functional recovery (13, 14). In recent years, multiple studies have provided direct evidence supporting this assertion. A qualitative study of elderly stroke patients found that active information-seeking behavior was significantly associated with higher self-efficacy and healthier behaviors, affirming the positive role of health information in secondary stroke prevention (15). Another multicenter cross-sectional study demonstrated that patients who effectively accessed and understood health information during rehabilitation achieved significantly superior self-management capabilities and functional independence scores. The health behavior decisions facilitated by this effective information access substantially enhanced patients’ quality of life and long-term prognosis outcomes (16). Therefore, research on health information-seeking behavior and health behavior decision-making in stroke patients can help solve health behavior management problems, and it has particular theoretical value and practical significance for implementing precise behavioral interventions and improving individual health outcomes.

However, the relationship between health Information-seeking behavior and behavioral decision-making in stroke patients remains underexplored. Furthermore, previous studies have evaluated patients’ health information-seeking behaviors based on scale scores, ignoring inter-individual heterogeneity (17, 18) and limiting themselves to a single variable or relationship between variables on the other, which deviates from the complex cognitive patterns of individuals in real life (19). Latent profile analysis (LPA), as an individual-centred classification technique (20), can identify patient subgroups exhibiting distinct health information-seeking behaviors. Based on this, healthcare professionals can move beyond one-size-fits-all educational approaches. By designing and implementing tailored interventions that address the behavioral characteristics of different subgroups, they can better match specific patient needs. This facilitates more effective translation of health information into sustained health behavior decisions.

Therefore, the present study employed latent profile analysis to investigate the latent characteristics of health information access behaviors among stroke patients and their relationship with health behavior decision-making, aiming to provide a robust evidence base for targeted behavioral interventions, improve patients’ access to health information. We believe that achieving more effective and personalized patient education will significantly enhance long-term clinical outcomes, including improved functional independence and reduced risk of stroke recurrence.

## 2 Methods

### 2.1 Study design, participants, and ethics

This cross-sectional study utilized a convenience sampling method to recruit 311 stroke patients who met the inclusion and exclusion criteria from January to May 2025 from the neurology, neurosurgery, and cerebrovascular disease departments of two tertiary-level A hospitals in Lanzhou City, serving as the survey subjects. Inclusion criteria comprised of patients who: (1) Meeting stroke diagnostic criteria (21); (2) patients aged ≥18 years old; (3) are conscious and in stable condition and able to complete the questionnaire survey either independently or with the assistance of the researchers; (4) signing an informed consent form to participate in this voluntarily The patients signed the informed consent and voluntarily participated in the study. Exclusion criteria included patients: (1) those with severe psychiatric disorders or aphasia; (2) those with severe physical diseases such as cardiac, pulmonary, and renal diseases; (3) those who are participating in other studies. Based on Sinha’s recommendation, a minimum of 50 subjects per subgroup was needed for accurate model fit in LPA (22). Since there were three profiles in this study, the required sample size should have been at least 150, considering a 20% inefficiency rate. Therefore, the minimum sample size needed was 188. A total of 330 questionnaires were distributed in this study. After excluding invalid responses due to irregular completion patterns, 311 valid questionnaires were recovered, with a valid recovery rate of 94.2%. The sample size was adequate for LPA-based analysis under these conditions. This study has been approved by the Ethics Committee of the Second Hospital of Lanzhou University (Approval No.2025A-110). The studies were conducted in accordance with the local legislation and institutional requirements. The participants provided their written informed consent to participate in this study.

### 2.2 Survey tools

#### 2.2.1 Demographic characteristics

The questionnaire developed by the research team through a comprehensive review of the literature and in conjunction with the research objectives and study content of this study, encompassed social demographic data (such as age, gender, literacy level, region of residence, marital status, personal monthly income, medical payment method, primary caregiver) as well as disease-related information including type of stroke, whether it was the first stroke, whether it was a smoking and drinking habit, whether it was comorbid with other chronic diseases, the degree of neurological deficits and recovery (MRS score), and self-care ability (Barthel Index).

#### 2.2.2 Health information-seeking behavior scale

The Health Information-seeking Behavior Scale, developed initially by Zamani et al. (23) and subsequently revised by Sun et al. (24). It has been widely adopted among patients with chronic diseases, comprises 43 items categorized into four dimensions: attitude towards health information-seeking (6 items), information demands (14 items), information sources (15 items), and barriers to acquiring health information (8 items) in the Chinese version. Using the Likert 5-level scoring method, one is very unimportant, and five is very important. The total score ranged from 0 to 215 points, with higher scores indicating a higher level of patients’ health information-seeking behavior. This scale has been validated in Chinese stroke patients. It demonstrates good reliability (25), with a Cronbach’s alpha coefficient of 0.90. In our study, Cronbach’s *α* coefficient was 0.87.

#### 2.2.3 Behavioral decision-making scale for stroke patients

The Behavioral Decision-making Scale for Stroke Patients was developed by Beilei Lin et al. (26). Specifically designed for stroke patients in China and widely used in this population (26). This scale comprises 30 items distributed across four dimensions: behavior change motivation (10 items), behavior change intention (9 items), decision-making factors (5 items), and decision-making balance (6 items). Each item was rated on a Likert 5-level scale, ranging from “strongly disagree” to “strongly agree,” with scores ranging from 0 to 4 points. The total score ranged from 0 to 150 points, with higher scores indicating a greater level of behavioral decision-making on behalf of stroke patients and facilitating the stimulation of healthy behavioral decision-making and the production of healthy behaviors. The scale’s reliability was good, with a Cronbach’s *α* coefficient of 0.934. In our study, Cronbach’s α coefficient was 0.765.

### 2.3 Data collection method

In this study, the survey subjects were screened in strict accordance with the inclusion and exclusion criteria, with questionnaires administered face-to-face by the trained investigators, and the purpose of the study, content, and filling requirements were explained in detail to the study subjects before the survey to obtain their consent of the patients. For participants with lower educational attainment and older adults, all questionnaire items were read aloud, asked, and recorded by the researcher on a question-by-question basis. Questionnaires were filled out on the spot and recalled for inspection. Data were double-entered and double-checked.

### 2.4 Statistical method

Mplus 8.3 was used for latent profile analysis of HISB in stroke patients. By using the mean values of the four-dimensional scores of the HISB scale as the manifest indicator, LPA was performed to fit models with 1 to 5 profiles sequentially. Model fit indices included: Akaike information criterion (AIC), Bayesian information criterion (BIC), and adjusted BIC (aBIC), as well as Entropy, Lo–Mendell–Rubin likelihood ratio (LMR), and Bootstrap likelihood ratio test (BLRT). Lower values of AIC, BIC, and aBIC indicated a better fit, while an Entropy value closer to 1 indicated a more precise classification. LMR and BLRT (*p* < 0.05) suggested that the k models were superior to the k-1 models.

SPSS software (version 27.0) was used to analyze the data. Quantitative data conforming to normal distribution were described by mean ± standard deviation ( $\bar{x}$ ±s), and qualitative data were described by frequency and percentage (%). One-way analysis was performed by chi-squared test, one-way analysis of variance (ANOVA) was used for comparison between groups of quantitative data, and unordered multicategorical logistic regression analysis was used to analyze influencing factors, with a significance level set at *α* = 0.05. In addition to *p*-values, effect sizes and 95% confidence intervals (CIs) were reported to quantify the magnitude and precision of the observed effects. The regression model adjusted for all statistically significant variables (*p* < 0.05) in the univariate analysis. This included demographic, clinical, and disease-related factors to control for potential confounding effects.

## 3 Results

### 3.1 Results of potential profiling of HISB in stroke patients

Based on the four dimensions’ scores of the health information-seeking behavior of stroke patients, divided into 1 to 5 potential profile models, Table 1 illustrates the possible profiles of the health information-seeking behavior of stroke patients. With the increase in the number of profiles, the values of AIC, BIC, and aBIC gradually decreased, and when three profiles were retained, the score of the Entropy indicator was 0.952. The LMRT and BLRT were statistically significant. Considering the accuracy and parsimony of the model by combining all indicators, model 3 was the best-fitting model, so the health information-seeking behavior of stroke patients was divided into three potential profiles.

**Table 1:** Fitting index for the latent profile model of the health information-seeking behavior in stroke patients.

Model	AIC	BIC	aAIC	Entropy	LMR (*P*)	BLRT (*P*)	Classification number
1	1566.320	1607.458	1572.570				1.000
2	1399.315	1459.152	1408.405	0.983	0.000	0.000	0.800 0.199
3	1209.266	1287.802	1221.198	0.952	0.000	0.000	0.591 0.210 0.199
4	1184.721	1281.946	1199.483	0.861	0.179	0.000	0.472 0.199 0.138 0.189
5	1160.139	1276.073	1177.752	0.872	0.148	0.000	0.434 0.199 0.385 0.173 0.154

AIC, The Akaike information criterion; BIC, Bayesian information criterion; LMRT, Lo–Mendell–Rubin adjusted likelihood ratio test.

### 3.2 Nomenclature and characterization of potential profiles of HISB in stroke patients

**Figure 1:**
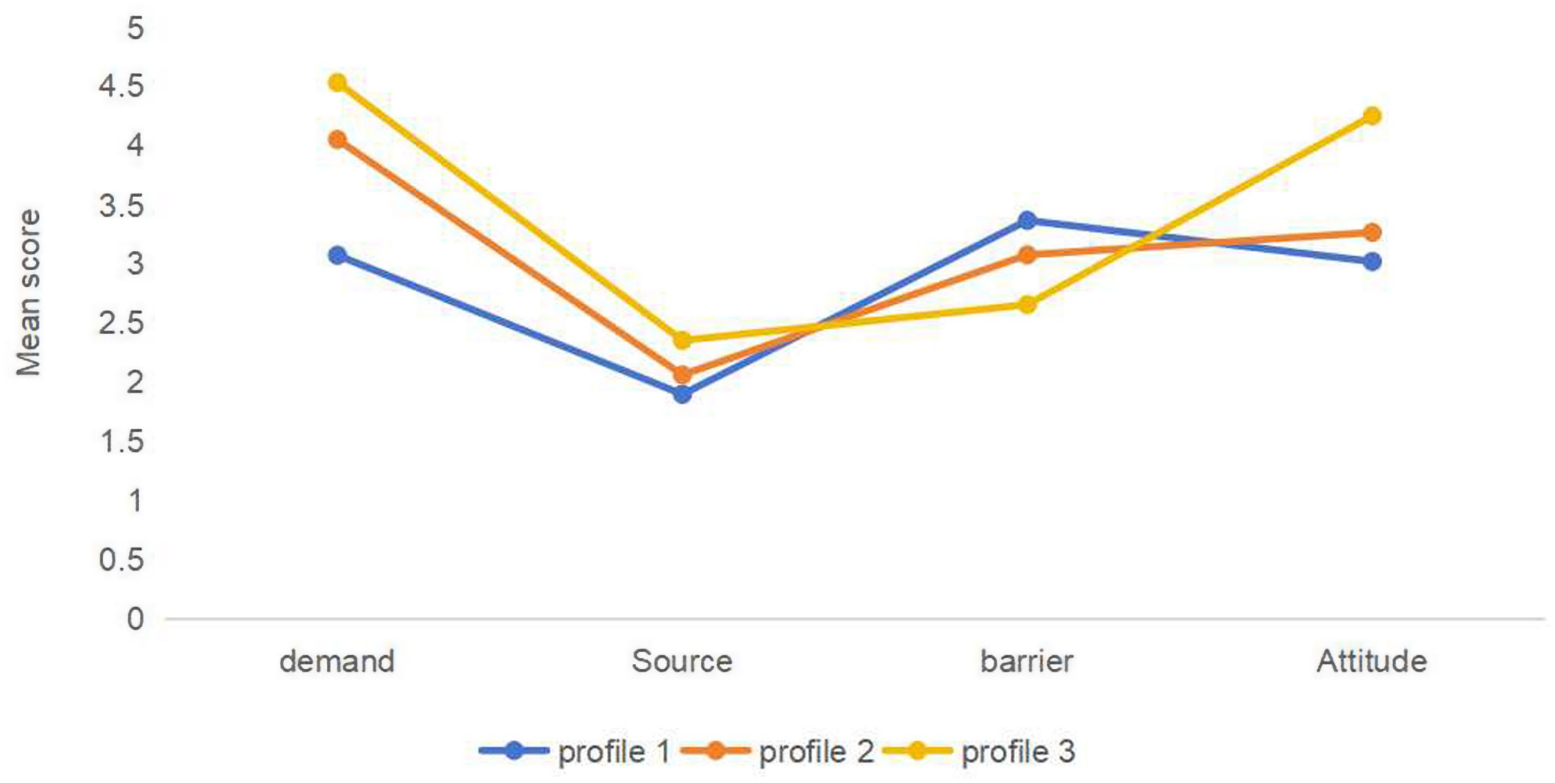
Potential profiles of health information-seeking behavior of stroke patients.

According to the scores on the four dimensions of the Health Information-seeking Behavior Scale (HISB) for the three profiles of stroke patients, it is clear that they showed different response characteristics, as shown in Figure 1. Stroke patients in profile 1 had low scores on the dimensions of information-seeking demands, sources, and attitudes, and the highest scores on the dimension of barriers to access. Accordingly, this category was named the “low demand and high barrier negative group,” with 62 cases (19.9%). Profile 2 had moderate scores in all dimensions, so it was named the “moderately balanced group,” with a total of 183 cases (59.1%). Stroke patients in profile 3 had the highest scores in information-seeking demands, sources, and attitudes, and the lowest in the dimensions of barriers to information-seeking. Thus, this category was termed the “high demand and low barrier positive group,” with 66 cases (21%), as shown in Table 2.

**Table 2:** Comparison of HISB scores in stroke patients with different potential profiles.

Variable	Low demand and high barriers negative group	moderately balanced group	High demand and low barriers positive group	*F*	*P*	*η^2^*
Information demands	3.07 ± 0.16	4.05 ± 0.15	4.53 ± 0.19	2.851	<0.001	0.082
Information sources	1.90 ± 2.86	2.06 ± 0.28	2.35 ± 0.25	1.834	0.014	0.054
Barriers to acquiring health information	3.36 ± 0.44	3.08 ± 0.52	2.65 ± 0.45	1.902	0.011	0.057
Attitude toward health information-seeking	3.02 ± 0.44	3.26 ± 0.42	4.26 ± 0.39	1.799	0.028	0.052
The total score of HISB	116.61 ± 5.65	131.83 ± 7.72	145.56 ± 5.56	8.005	<0.001	0.215

F, ANOVA for continuous variables; *p* < 0.05; η^2^, eta-squared.

### 3.3 Univariate analysis of factors influencing potential HISB profiles in stroke patients

Differences in health information-seeking behaviors among different potential profiles of stroke patients were statistically significant (*p* < 0.05) in the distributions of age, region of residence, literacy level, monthly personal income, method of payment for medical care, type of stroke, Barthel Index, and MRS scores across the three potential profiles, as shown in Table 3.

**Table 3:** Univariate analysis of factors influencing the potential profile of HISB in stroke patients.

Variables		Low demand and high barriers negative group (62)	Moderately balanced group (183)	High demand and low barriers positive group (66)	*χ^2^*	*P*
Sex	Male	32 (51.6)	116 (63.4)	45 (68.2)	4.06	0.130
	Female	30 (48.4)	67 (36.6)	21 (31.8)		
Age (years)	≤59	10 (16.1)	47 (25.7)	34 (51.5)	27.71	<0.01
	60–74	38 (61.3)	104 (56.8)	28 (42.4)		
	≥75	14 (22.6)	32 (17.5)	4 (6.1)		
Residence	Countryside	34 (54.8)	81 (44.3)	19 (28.8)	14.39	0.010
	Town	14 (22.6)	48 (26.2)	14 (21.2)		
	City	14 (22.6)	54 (29.5)	33 (50.0)		
Marital status	Married	41 (66.1)	142 (77.6)	48 (72.7)	14.67	0.230
	Single/Divorced/Widow	21 (33.8)	41 (22.4)	18 (27.3)		
Education level	Primary school and below	35 (56.5)	62 (33.9)	5 (7.6)	62.70	<0.010
	Junior middle school	15 (24.2)	68 (37.2)	27 (40.9)		
	High or vocational school	8 (12.8)	44 (24.0)	13 (19.7)		
	College and above	4 (6.5)	9 (4.9)	21 (31.8)		
Monthly income (RMB)	≤3,000	35 (56.5)	48 (26.3)	10 (15.2)	39.70	<0.01
	3,000–5,000	17 (27.4)	63 (34.4)	15 (22.7)		
	>5,000	10 (16.1)	72 (39.3)	41 (62.1)		
Medical insurance type	Employee medical insurance	5 (8.1)	31 (16.9)	20 (30.3)	20.16	0.000
	Medical insurance for urban residents	23 (37.1)	64 (35)	30 (45.4)		
	New rural cooperative medical care	34 (54.8)	88 (48.1)	16 (24.3)		
Primary caregiver	Own	16 (25.8)	38 (20.7)	23 (34.8)	11.78	0.670
	Spouse	15 (24.2)	66 (36.1)	21 (31.8)		
	Children	30 (48.4)	79 (43.2)	23 (35)		
Whether it was the first stroke	Yes	38 (61.3)	122 (66.7)	47 (71.2)	1.41	0.490
	No	24 (38.7)	61 (33.3)	19 (28.8)		
Type of stroke	Haemorrhage	11 (17.7)	25 (13.7)	20 (30.3)	9.10	0.010
	Ischemic	51 (82.3)	158 (86.3)	46 (69.7)		
Comorbidity with other chronic diseases	Yes	17 (27.4)	99 (54.1)	49 (74.2)	28.33	< 0.010
	No	45 (72.6)	84 (45.9)	17 (25.8)		
Smoking and drinking	Yes	12 (19.4)	42 (23.0)	31 (47.0)	16.57	< 0.01
	No	50 (80.6)	141 (77.0)	35 (53.0)		
Barthel’s index (score)	100	2 (3.2)	8 (4.4)	12 (18.2)	17.20	0.002
	61–99	45 (72.6)	143 (78.1)	42 (63.6)		
	41–60	15 (24.2)	32 (17.5)	12 (18.2)		
MRS scores	0	2 (3.2)	4 (2.2)	4 (6.1)	29.50	< 0.010
	1	0 (0)	19 (10.4)	18 (27.3)		
	2	33 (53.3)	78 (42.6)	24 (36.4)		
	3	26 (41.9)	73 (39.9)	19 (28.8)		
	4	1 (1.6)	9 (4.9)	1 (1.4)		

χ^2^, Chi-squared test; *p* < 0.05.

### 3.4 Multifactorial analysis of factors influencing potential HISB profiles in stroke patients

An unordered multicategorical logistic regression was performed with the three potential profiles of health information-seeking behavior of stroke patients as dependent variables (low demand and high barrier negative group = 1, moderately balanced group = 2, high demand and low barrier positive group = 3) with variables that were statistically significant in the univariate analysis as independent variables. The independent variables were assigned: literacy level: Primary school and below = 1, Junior middle school = 2, High or vocational school = 3, college and above = 4; personal monthly income: <3,000 = 1, 3,000–5,000 = 2, >5,000 = 3; and whether or not to comorbid with other chronic diseases: yes = 1, no = 2. The results showed that age, literacy level, personal monthly income, and whether or not to be comorbid with other chronic diseases were the different potential profiles of the significant influencing factors (*p* < 0.05), see Table 4.

**Table 4:** Multivariate logistic regression analysis on the latent profile of HISB in stroke patients.

Variable	Low demand and high barrier negative group^a^				Moderately balanced group^a^			
	*β*	*P*	OR	95%CI	*β*	*P*	OR	95%CI
Constant term	−4.798	0.023			−5.413	0.002		
Age	0.077	0.010	1.080	(1.018, 1.146)	0.094	<0.050	1.098	(1.046, 1.152)
Education level Primary school or below	0.403	0.009	1.496	(1.104, 2.028)	0.005	0.677	1.051	(0.829, 1.332)
Monthly income > 5,000	0.052	0.781	1.051	(0.738, 1.497)	−1.442	0.029	0.237	(0.065, 0.860)
A combination of other chronic diseases	−2.325	< 0.001	0.098	(0.036, 0.264)	−1.320	0.002	0.267	(0.115, 0.621)

The high demand and low barrier positive group was used as the reference category. OR, odds ratio; CI, confidence interval.

### 3.5 Relationship between HISB latent profiles and health behavior decision-making in stroke patients

The results of ANOVA showed that the differences between different potential profiles of HISB in stroke patients were statistically significant in motivation to change behavior, intention to change behavior, decision-making factors, and decision-making balance (*p* < 0.001), as shown in Table 5.

**Table 5:** Behavioral decision-making scores for stroke patients with different potential profiles.

Variable	Low demand and high barrier negative group	Moderately balanced group	High demand and low barrier positive group	*F*	*P*	*η^2^*
The total score of behavioral decision-making	97.13 ± 10.31	106.41 ± 8.17	114.74 ± 8.17	66.42	<0.001	0.382
Motivation for behavior change	34.44 ± 3.70	37.42 ± 2.92	41.38 ± 4.47	65.43	<0.001	0.379
Behavior change intention	30.35 ± 3.45	32.91 ± 2.61	34.48 ± 4.18	27.69	<0.001	0.215
Decision-making factors	15.90 ± 1.85	17.30 ± 2.12	18.52 ± 2.63	22.67	<0.001	0.198
Balanced decision-making	16.44 ± 2.83	18.79 ± 3.19	20.36 ± 3.51	24.56	<0.001	0.205

F, ANOVA for continuous variables; *p* < 0.05; η^2^, eta-squared.

## 4 Discussion

### 4.1 Heterogeneity in health information-seeking behavior among stroke patients

Health information-seeking behavior, also known as health information query behavior and health information search behavior, refers to individuals seeking information about health, risk, disease, and health protection in specific events or situations (27). In this study, we found that the total score of health information-seeking behavior of stroke patients was (131.71 ± 11.58), which was at a low level, slightly lower than the results of the related study by Yufan et al. (25). This may be related to the fact that most of the study subjects included in this study were elderly patients in the more economically backward areas of Northwest China, and that due to the limitations of physical function and cognitive level, the elderly have relatively limited ability and channels to obtain information and low comprehension of information (28). Health information-seeking behaviors among stroke patients exhibit group heterogeneity. Among them, 21.1% of the patients’ health information-seeking behavior was high. The patients had a strong demand for disease knowledge, rehabilitation skills, and other information, and a positive attitude toward acquiring knowledge; and the barriers to accessing information sources were relatively low. The patients alleviated the uncertainty of the disease by seeking a high demand for information to improve their self-efficacy, which is consistent with the viewpoint that “a sense of information mastery promotes proactive participation” in the theory of health empowerment (29). This is consistent with the idea that “information mastery promotes active participation” in health empowerment theory (29). It may be related to the fact that patients in this category are mainly concentrated in the middle-aged (≤59 years old) and higher education (high school/secondary school and above) groups, which generally have a higher level of health literacy and a greater ability to learn and cognitively perceive specialised medical knowledge. The majority of patients (59.9%) were at the moderately balanced level, which may be because the patients in this profile were concentrated in the age group of 60–74 years old, and their education was mainly in junior high school, so their knowledge learning ability and seeking of information and cognitive comprehension were limited. 19.9% of patients were in the low-demand, high-barrier, and negative group, characterised by negative attitudes toward the disease and a significant limitation in their demand. This group may have been affected by cognitive bias toward the disease or inadequate health literacy due to their old age; however, their health literacy was generally high. They may be affected by disease cognitive bias or health literacy, and have high barriers to information-seeking due to their age, low literacy level, and a single source of information seeking channels, suggesting that clinical healthcare professionals need to identify the differences in the nursing care needs of different categories of the population as early as possible and implement personalized nursing care interventions to improve the level of patients’ health information-seeking behaviors.

### 4.2 Factors influencing different potential profiles of HISB in stroke patients

The results of this study indicate that older and less educated patients are more likely to be categorized into the low-demand, high-barrier negative group. Analyzing the reasons, younger patients tend to have higher health awareness and treatment compliance. They are more active in communicating with the outside world to obtain help and enhance their understanding of disease information. Older adults often experience age-related cognitive and psychological changes, manifested as declines in work capacity, memory, and information processing speed, which can diminish their ability to absorb complex health information. Additionally, elderly patients may exhibit lower self-efficacy and greater technophobia when navigating the healthcare system, further inhibiting proactive information-seeking behaviors (30). Patients with higher literacy levels are more inclined to proactively acquire health information. First, their educational background furnishes them with superior cognitive resources, including enhanced health literacy and comprehension of medical terminology, which lowers the cognitive barrier to processing complex information (31). Beyond this, Education can foster forward-thinking health concepts and enhance self-efficacy, thereby stimulating the intrinsic motivation to manage one’s health proactively (32). In addition, the advantage of digital literacy brought by an educational background also increased their efficiency in utilizing smart devices and reducing technical barriers to information access (33). It is suggested that healthcare professionals should pay attention to the level of information-seeking behaviors of elderly and low-education patients, guide their families to help them communicate with healthcare professionals about their conditions and participate in medical decision-making, and adopt an easy-to-understand approach to inform patients about their conditions and treatments to make it easier for them to understand.

The results of this study showed that patients with a personal monthly income of more than 5,000 yuan were more likely to be categorized as high-demand, low-barrier positive group, probably because the higher the income level, the higher the level of economic capital, educational advantages, social network, and medical accessibility (34). Higher income provides access to broader social networks, which serve as channels for obtaining health information. Furthermore, income inequality creates disparities in exposure to health information, with higher-income individuals more frequently engaging with health-literate social circles and health management programs. Beyond this tangible resource, greater economic resources typically experience enhanced psychological security, which reduces the cognitive burden associated with health concerns and frees up mental resources for information seeking (35). Therefore, healthcare professionals should pay attention to the assessment of patients’ economic level, pay attention to low-income patients’ demands for health information and access to health information, develop concise health education tools covering disease awareness and rehabilitation guidance, and instruct them to master basic information retrieval skills and enhance patients’ ability to obtain health information by optimizing the comprehensibility and ease of operation of the educational content.

The results of this study showed that the greater the likelihood that patients with comorbid chronic diseases, such as hypertension and diabetes, would be categorized as moderately well-balanced and high-demand low-barrier active, similar to the results of Wang et al. (36). The reason may be that, First, the comorbid state forces patients to manage multiple health problems simultaneously, and the demand for health information is more urgent, which leads to active interdisciplinary access to health-related information to optimize decision-making. Second, patients with chronic diseases have developed habits of regular monitoring and medication adjustments (37). Their long-term management experience enhances their information efficacy. Their self-efficacy supports the screening and application of complex information, while stroke events further catalyze patients’ demand for systematic knowledge (38). Third, patients with comorbidities of other chronic diseases were more likely to have access to professional guidance due to frequent doctor visits, and the advantage of resource utilization was more pronounced, which could significantly reduce their barriers to information seeking (39). This suggests that healthcare workers should dynamically assess the changes in information demands of different patients, pay more attention to patients with lower levels of health information-seeking behavior, and strengthen publicity and guidance. For patients with higher levels of health information-seeking behavior, systematic and interdisciplinary integrated knowledge services should be provided.

### 4.3 Relationship between different potential profiles of HISB and health behavior decisions in stroke patients

This study revealed that stroke patients with varying potential profiles demonstrated significant differences in health behavior decision-making and related dimensions. Patients with high-demand, low-barrier positive profiles showed optimal health behavior decision-making ability, demonstrated higher initiative and adaptability in health behavior change, and perceived fewer external barriers, which may be attributed to the fact that patients with strong demand are more inclined to explore information proactively (40), and those with positive attitudes are more willing to invest time and energy in understanding the information, which in turn translates into concrete actions (41). This positive information-seeking tendency leads to a greater likelihood of forming clear behavioral intentions and translating them into actual actions, which is conducive to the prognosis of the disease (42). In contrast, negative patients with low demands and high barriers may be limited by insufficient motivation or limited sources of information seeking, which interferes with the patient’s comprehensive assessment of decision-making, thus affecting the quality of health behavioral decision-making and facing greater resistance to behavioral change, and consequently, involving the implementation of the overall health behaviors (43). Moderately balanced patients fall between extremes, and multifactorial dynamics may influence their behavioral decisions. This difference in typology underscores the demand for individualized strategies in clinical interventions. Clinical staff can focus on continuously optimizing information supply and providing positive feedback reinforcement for patients with high demand and low barriers.

In contrast, patients with low demand and negative obstacles require strengthening motivational and cognitive interventions, enhancing their health information sensitivity through motivational interviews and health writing, and systematically addressing challenges at the environmental and social support levels. The systematic reduction of ecological and social support barriers. In this way, we can more effectively improve the health information-seeking behavior of stroke patients, stimulate health behavior decision-making, and promote the formation and maintenance of health behaviors.

### 4.4 Implications for clinical practice and health policy

By identifying three distinct latent profiles of HISB, this study provides critical evidence for developing targeted patient education and support strategies. Clinically, interventions must be tailored to each profile: For the high demand and low barrier positive group, healthcare professionals should deliver systematic, in-depth, and multidisciplinary health information to satisfy their strong initiative and reinforce their already optimized health behavior decision-making capacity. For the moderately balanced group, standardized health education programs centered on foundational knowledge and skill development are essential to effectively enhance their information efficacy. Most critically, interventions for the low demand and high barrier negative group, typically older, less educated, and lower-income patients, should combine motivational interviewing to overcome negative attitudes, simplify health materials for improved comprehension, and actively engage family or caregivers to bridge the information gap (44). To systematically support these tailored interventions, future policy measures could include integrating health literacy assessments into routine clinical care, developing tiered health education resources, enhancing training for primary healthcare workers, and promoting the establishment of integrated information support networks linking hospitals, communities, and households (45). Furthermore, given that factors like age, education level, income, and comorbidities significantly influence health information-seeking patterns, screening for these characteristics upon admission enables early identification of high-risk patients. Ultimately, behavior-based interventions that remove barriers and cultivate proactive information-seeking mindsets will effectively promote healthier behavioral decisions, thereby accelerating functional recovery and reducing stroke recurrence rates among stroke patients.

## 5 Limitations and future research

This study had some limitations that warrant consideration. Firstly, the reliance on self-reported measures is susceptible to reporting biases, such as social desirability and recall bias, which may affect the accuracy of the data on health information-seeking behavior and decision-making. Secondly, the cross-sectional design precludes the determination of causal relationships between the identified latent profiles, their predictors, and health behavior decision-making. Lastly, the relatively small sample size limits the representativeness of the findings and thus constrains the generalizability of the results. Future research should employ longitudinal or mixed-methods designs with multi-center, increase the sample size, randomized sampling strategies to validate the identified profiles, explore their stability over time, and establish causal pathways. Additionally, incorporating objective measures of health information-seeking could complement self-reported data and provide a more comprehensive understanding.

## 6 Conclusion

This study reveals significant group heterogeneity in health information-seeking behaviors among stroke patients and explores the relationship between their underlying profiles and health behavior decision-making. These findings can guide clinicians in designing precision education or intervention programs tailored to specific patient subgroups. Shifting clinical practice from standardized education to precision-targeted interventions, this strategy constructs an effective pathway for health information to guide rational decision-making, ultimately promoting the formation and maintenance of health.

## Data Availability

The original contributions presented in the study are included in the article/supplementary material, further inquiries can be directed to the corresponding author.
